# Supplementary material for: Investigating the influence of physiologically relevant hydrostatic pressure on CHO cell batch culture
Source: Sci Rep. 2021 Jan 8;11:162. doi: 10.1038/s41598-020-80576-8 (PMC7794228; doi:10.1038/s41598-020-80576-8)
Supplement: Supplementary file 1 — Supplementary Information 1. [file 41598_2020_80576_MOESM1_ESM.zip › S1/FLEX2/13Jan 30mmHg SS1 is C/Jan 12 Control.pdf]

===

Acq. Operator : SYSTEM   Seq. Line : 4
Sample Operator : SYSTEM
Acq. Instrument : HPLC-0039   Location : 4
Injection Date : 1/13/2019 5:37:31 PM   Inj : 1
Inj Volume : 100.000 µl
Sequence File : C:\Users\Public\Documents\ChemStation\1\Data\TK_CHO_IgG 2019-01-13 17-23-06\TK_CHO_IgG.S
Method : C:\Users\Public\Documents\ChemStation\1\Data\TK_CHO_IgG 2019-01-13 17-23-06\CHO_IgG_LC.M (Sequence Method)
Last changed : 12/4/2018 1:38:41 PM by SYSTEM

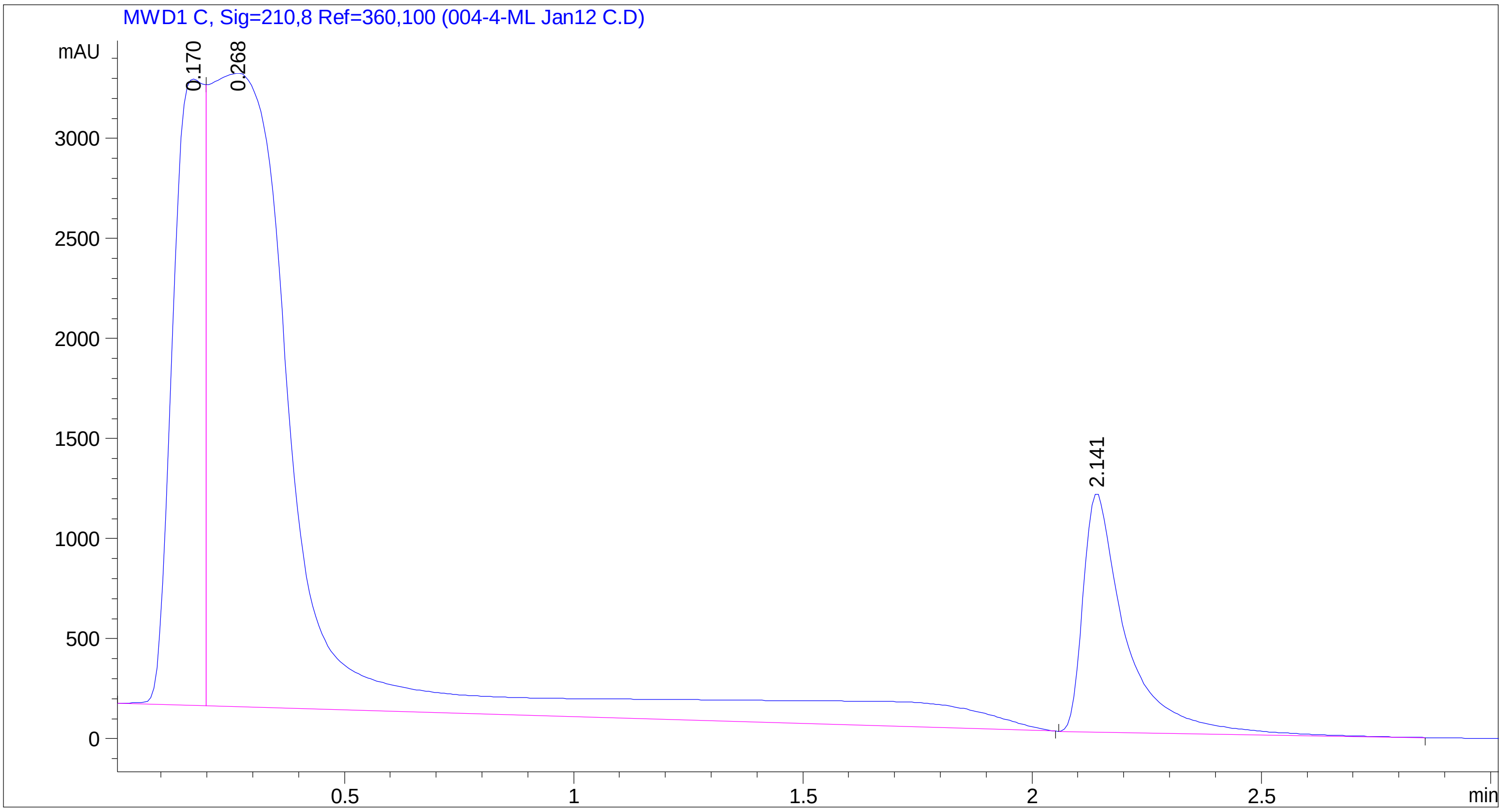

===

Area Percent Report

===

Sorted By : Signal
Multiplier : 1.0000
Dilution : 1.0000
Use Multiplier & Dilution Factor with ISTDs

Signal 1: MWD1 C, Sig=210,8 Ref=360,100

Peak #	RetTime [min]	Type	Width [min]	Area [mAU*s]	Height [mAU]	Area %
1	0.170	BV	0.0729	1.47279e4	3131.56299	21.8965
2	0.268	VB	0.2091	4.48044e4	3165.72192	66.6123
3	2.141	BBA	0.0935	7729.10693	1195.01575	11.4911

Totals : 6.72614e4 7492.30066

===

*** End of Report ***
